# Upper airway resistance during use of a laryngeal mask airway is flow-dependent and dominated by the laryngeal resistance

**DOI:** 10.1038/s41598-024-73844-4

**Published:** 2024-10-09

**Authors:** Johannes Hell, Axel Schmutz, Stefan Schumann

**Affiliations:** 1 Department of Anesthesiology and Critical Care, Medical Center, University of Freiburg, Hugstetter Str. 55, 79106 Freiburg, Germany; 2 Faculty of Medicine, University of Freiburg, Freiburg, Germany

**Keywords:** Laryngeal mask airway, Upper airway resistance, Laryngeal resistance, Combined resistance, Flow dependant resistance, Preclinical research, Biomedical engineering

## Abstract

During use of a laryngeal mask airway, resistance of the device and larynx contribute to the upper airway resistance. Detailed understanding of this combined resistance is crucial to support spontaneously breathing patients appropriately or to take the right measures during respiratory problems. However, their resistive behavior and which of these components determine the upper airway resistance predominantly, has not been characterized systematically. Pressure-flow-relationships of different sizes of a laryngeal mask airway, of a laryngeal model with vocal cord angles between 10° and 60° and of the combination of a laryngeal mask airway size 4 and the laryngeal model were measured. Results were fitted to the expanded Rohrer’s equation and resistances were calculated. The laryngeal mask airway and the laryngeal model showed a nonlinear flow-dependent resistive behavior. Decreasing size of the laryngeal mask airway, decreasing vocal cord angles, and increasing flow rates resulted in increased resistances (all *p* < 0.001). Resistance of the laryngeal mask and the laryngeal model added up to the combined resistance in a summative way, where the vocal cord angle determines 59–98% of the combined resistance in adults. The upper airway resistance during the use of a laryngeal mask airway is a summative resistance with a flow-dependent, nonlinear behavior. Upper airway resistance in adults is primarily determined by the vocal cord angle during use of a laryngeal mask.

**Supplementary Information:**

The online version contains supplementary material available at 10.1038/s41598-024-73844-4.

## Introduction

The popularity of the laryngeal mask airway (LMA), a device to connect the respirator to patients airways during mechanical ventilation, is due to its ease of use. Insertion is generally unproblematic and can be achieved without neuromuscular blockade. The risk of compromising the vocal cords or the trachea is reduced when compared to tracheal intubation^1^. The LMA provides an end-to-end airway connection, while being placed at the entrance of the trachea. This contrasts with a plug-in connection when a endotracheal tube (ETT), which has to be placed inside the patients trachea, is used^2^. The type of connection decisively influences the geometry of the airway device itself, its physical characteristics of air conduction, and its resistance. The resistance of the airway device influences the pressure drop over the device and the tracheal and intrapulmonary pressure in mechanically ventilated patients^3^ or the work of breathing (WOB) in spontaneously breathing patients^4^. In general, the internal diameter of the LMA is larger and the length is shorter than a corresponding ETT, resulting in a lower device resistance^5^. However, contrary to the ETT, the LMA does not bridge the larynx, so upper airway resistance consists of a combination of the resistance of the LMA and the resistance of the larynx^6^, mainly defined by the width of the glottic aperture, which varies with the angle of the vocal cords^7^. The misjudgment of the upper airway resistance during use of the LMA due to the lack of a precise knowledge of its composition clarifies the ambivalent results of previous investigations analyzing resistance^5,8–11^ or WOB^5,12,13^ of the LMA in comparison with the ETT to some extent. Furthermore, to compensate the additional WOB to overcome the resistance of the LMA in spontaneously breathing patients, a precise knowledge of the superimposed resistance is required and, if difficulties in mechanical ventilation occur or high inspiratory pressures are needed to ensure adequate ventilation, a fundamental understanding of the combined resistance is needed to take the best measures. Therefore, to gain a better understanding of the resistive behavior of the respiratory system during the use of an LMA, a systematic characterization of upper airway resistance as a combination of LMA resistance and laryngeal resistance and how they contribute to the combined resistance is needed.

The aim of our study was to investigate the pressure-flow-relationship of all sizes of a contemporary LMA and to characterize the combination of the resistances of the LMA and the larynx and their proportional contribution to the upper airway resistance. We hypothesised that (a) the resistances of the LMA, and larynx are nonlinear and flow-dependent, (b) the resistance increases with increasing flow, decreasing LMA size, and decreasing vocal cord angle (VCA), and (c) the resistances of the LMA and larynx add up to the combined resistance in a summative manner. Therefore, we systematically investigated the resistances of different sizes of LMAs, VCAs, and their combinations in a physical model.

## Methods

The experimental bench study was conducted from March 2022 to November 2022. To determine the resistance, we measured the pressure drop across eight different LMA sizes (LMA#1 to LMA#6, Table 1) of the laryngeal mask Ambu^®^AuraOnce™ (Ambu, Ballerup, Denmark) and the pressure drop across a physical model of the larynx with six different vocal cord angles (10°–60° in steps of 10°) during a sinusoidal flow pattern to generate varying flow rates. Furthermore, we measured the pressure drop across the combination of LMA#4 and the physical laryngeal model simulating the geometries of an adult patient (geometry data for the laryngeal model according to Filho et al.^14^), in whom LMA#4 would be used, with different VCAs.

**Table 1:** Overview of the investigated LMAs.

LMA size	#1	#1.5	#2	#2.5	#3	#4	#5	#6
Patient weight (kg)	< 5	5–10	10–20	20–30	30–50	50–70	70–100	> 100
Minimal internal diameter (mm)	5.2	7.3	8.6	8.5	8.5	9.6	10.6	11.3
Length of the airway (cm)	10.3	12.0	13.8	15.9	15.9	17.8	20.0	22.0

Overview of the different LMA-sizes and the recommended patient’s weight for use. Additionally minimal internal diameter and length of the airway were presented. Data was extracted of the user’s manual^17^.

### Experimental setup

**Fig. 1:**
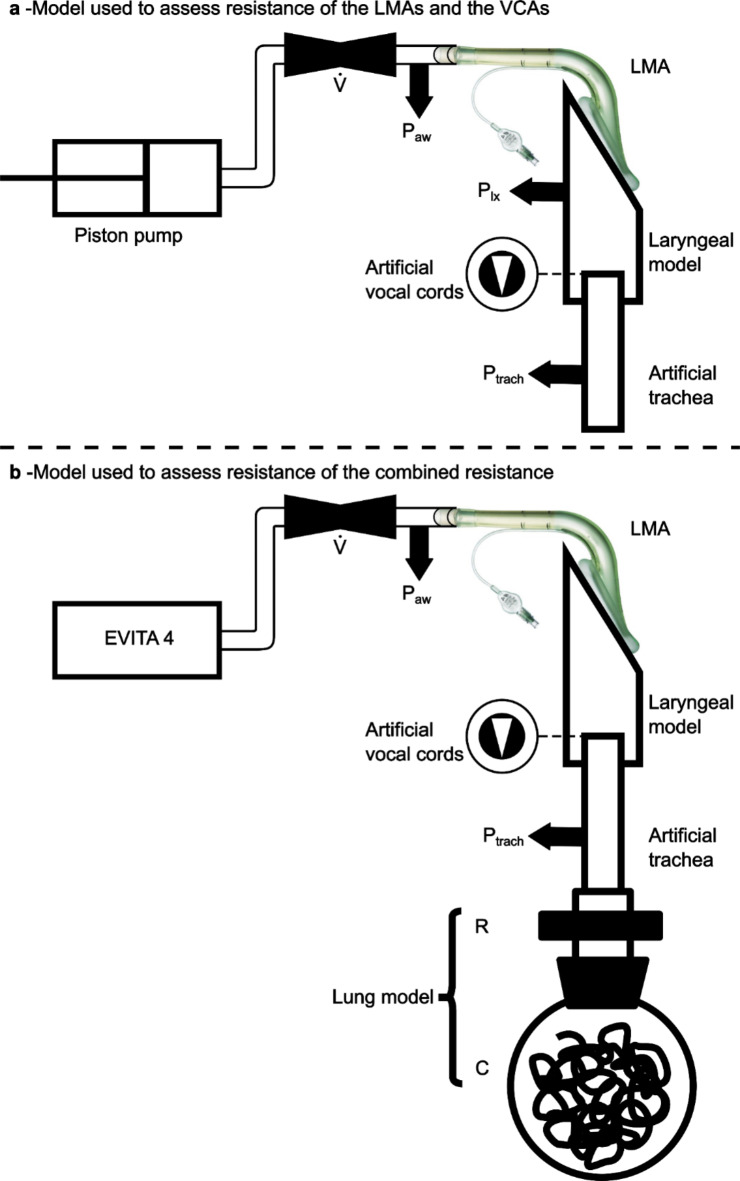
Experimental setups. (**a**) Setup used for measurement of the resistance of the different sizes of the laryngeal mask airways and the different vocal cord angles. A flow generator delivered a sinusoidal flow to the laryngeal mask airway (LMA), connected to the laryngeal model with the artificial vocal cords. V̇ indicates pneumotachograph. P indicates pressure transducers for airway pressure (P_aw_), laryngeal pressure (P_lx_) and tracheal pressure (P_trach_). (**b**) Setup used for measurement of the combined resistance (LMA#4 and the different vocal cord angles). A ventilator (EVITA 4) delivered pressure-controlled ventilation to the laryngeal mask airway (LMA), connected to the laryngeal model with the different vocal cord angles. Laryngeal model was connected to a heat-moisture exchange filter representing resistance (R). A glass bottle represented the compliance (C) of the physical lung model.

A sinusoidal flow was generated by a self-developed linear motor-driven piston pump with a frequency of 18 min^− 1^ and a peak flow rate of ± 2 l s^− 1^. The LMA was connected to the laryngeal model (Fig. 1a, Supporting Information Fig. S1a) designed using 3D-CAD-software (Autodesk Inventor Professional 2019, Autodesk, SanRafael, USA) and printed using polylactic acid (PLA, Ultimaker, Geldermalsen, Netherlands) with a 3D-printer (Ultimaker 3, Ultimaker, Geldermalsen, Netherlands). Construction drawings of the model are presented in the supplementary material (Supporting Information, Fig. S2–S5). The laryngeal model consisted of a contact surface for the LMA’s cuff and an artificial trachea with exchangeable vocal cords. The diameters of the modelled vocal cords and trachea corresponded to those of an adult female^14^. For LMA#1, LMA#1.5, and LMA#2, an additional adapter to connect the cuff to the contact surface was built. The LMAs were connected to the laryngeal model with adhesive tape (Supporting Information Fig. S1c + d). Fixation was adjusted until no audible leakage was detectable and expiratory tidal volume was equivalent to inspirator tidal volume during ventilation of the lung model. The artificial trachea was open to atmosphere. The pressure-flow-relationships of each LMA and each VCA of the laryngeal model were measured in five repetitions, including 12 consecutive sinusoidal flow cycles. Between the repetitions, complete disassembly and reassembly of the laboratory setup and calibration of all sensors were performed. Flow rates were measured using a pneumotachograph (Fleisch Type 2, Dr. Fenyves & Gut, Hechingen, Germany) and pressures were measured using piezoresistive differential pressure transmitters (Type 2, Special Instrument, Nördlingen, Germany). Airway pressure (P_aw_) was measured in immediate proximity to the LMA, and laryngeal pressure (P_lx_) between the end of the LMA and the vocal cords. Tracheal pressure (P_trach_) was measured 10.8 cm below the artificial vocal cords to avoid measuring errors due to turbulent flow. The pressure drop across the LMA (ΔP_LMA_) was defined as the difference between P_aw_ and P_lx_, and the pressure drop across the glottis (ΔP_GL_) was defined as the difference between P_lx_ and P_trach_. To investigate the combined resistance of LMA#4 and the different VCAs, pressure-controlled ventilation (PCV) was applied (respiratory frequency 12 min^− 1^, peak inspiratory pressure 15 cmH_2_O or 20 cmH_2_O, inspiratory to expiratory time ratio 1:2, positive end-expiratory pressure 5 cmH_2_O, O_2_ concentration 21%) with an intensive care ventilator (Evita 4, Dräger, Lübeck, Germany). The air was conducted via the LMA and the laryngeal model to a physical lung model, which consisted of a 56 l glass bottle representing respiratory system compliance, and a heat and moisture exchange filter (TwinStar, Dräger, Lübeck, Germany) representing the resistance of the lung (Fig. 1b, Supporting Information Fig. S1b). The glass bottle was filled with copper wool to provide isothermal conditions. Each composed resistance was measured for 12 consecutive breaths in six repetitions. Between the repetitions, complete disassembly and reassembly of the laboratory setup and calibration of all sensors were performed. The pressure drop across the combined resistance (ΔP_CR_) was determined as the difference between the P_aw_ and P_trach_.

All analogue signals were digitalized with a sampling rate of 200 Hz.

## Calculations

The pressure-flow-relationships were characterized by applying an expanded Rohrer’s equation accounting for inertance^15,16^

$$\:\varDelta\:p={K}_{1}\cdot\:\stackrel{}{\dot{V}}+{K}_{2}\cdot\:{\stackrel{}{\dot{V}}}^{2}+I\cdot\:\stackrel{}{\ddot{V}}$$

where $\:\varDelta\:p$ is the pressure-difference across the resistance being tested, $\:{K}_{1}$ and $\:{K}_{2}$ are Rohrer’s resistance coefficients, $\:I$ is inertance, $\:\dot{V}$ is flow rate and $\:\ddot{V}$ is volume acceleration. Multilinear regression analysis was used to determine Rohrer’s coefficients $\:{K}_{1}$ and $\:{K}_{2}$. The flow-dependent resistance $\:R\left(\dot{V}\right)$ was then calculated by dividing the resistance part of Rohrer’s equation by the flow rate as follows:

$$\:R\left(\dot{V}\right)=\frac{\varDelta\:p}{\dot{V}}==\frac{{K}_{1}\cdot\:\stackrel{}{\dot{V}}+{K}_{2}\cdot\:{\stackrel{}{\dot{V}}}^{2}}{\dot{V}}={\frac{{K}_{1}\cdot\:\dot{V}+{K}_{2}\cdot\:\dot{V}\:\cdot\:\dot{V}}{\dot{V}}=K_{1}}+{K}_{2}\cdot\:\dot{V}$$

For allowing quantitative comparisons, the resistances for an exemplary flow rate of 1 l s^− 1^ were determined.

To evaluate, if the combined resistance follows the expected physical principle of summation of the single resistances, measured combined resistance was calculated using the measured ΔP_CR_, and was compared with the sum of the single corresponding resistances of LMA and vocal cords, determined during the experiment. Adherence was classified as marker of quality of the measured results and the physical model.

## Statistics

Data are presented as mean ± SD or mean (95% Confidence Interval) if not indicated otherwise. To ensure high quality of the fit of Rohrer’s equation to the recorded data, correlation coefficients were calculated. For statistical evaluation of the effect of LMA size, VCA, and flow rate on resistance, the resistance was analyzed in a flow range between 0 and 1.5 l s^− 1^ in steps of 0.25 l s^− 1^. We used a linear mixed model with LMA size or VCA and flow as fixed effects, where flow was determined as the repeated effect value. Compound symmetry with correlation parameterization was used as estimated covariance structure. The Bonferroni correction was used for post-hoc pairwise comparisons. To compare the resistance between different flow rates and different LMA sizes, the differences of the mean resistances were calculated. Inspiratory and expiratory resistances at a flow of 1 l s^− 1^ were compared for each LMA size and each VCA with a t-test for paired samples. Statistical significance was assumed at *p* < 0.05. Data were analyzed using SPSS (version 27.0, Chicago, Illinois, USA).

## Results

**Fig. 2:**
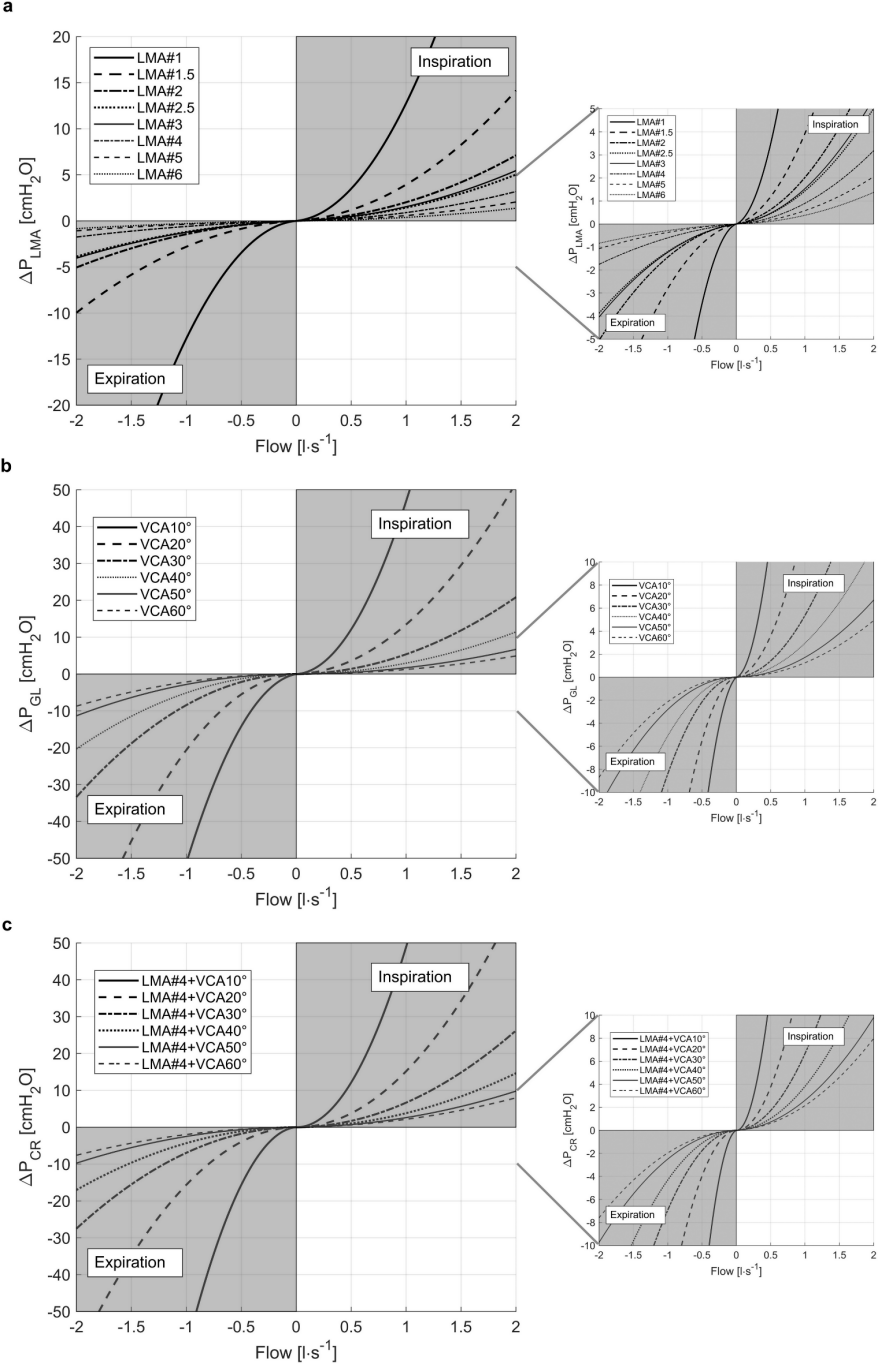
Flow-dependent pressure-drop. (**a**) Flow-dependent pressure-drop (ΔP_LMA_) of the different sizes (#) of laryngeal mask airways (LMA), (**b**) of the glottis model (ΔP_GL_, B) at different vocal cord angels (VCA) and (**c**) of the combination of a laryngeal mask airway size 4 (LMA#4) and the different VCAs (ΔP_CR_) with the corresponding zoomed view on the right. Inspiration was defined as positive flow rate and expiration as negative flow rate.

All LMAs, VCAs, and the combination of LMA#4 and VCAs showed nonlinear pressure-flow-relationships (Fig. 2).

The nonlinear pressure-flow-relationships of all tested objects could be well described by Rohrer’s equation (all R^2^ > 0.9). By analogy to Rohrer’s coefficients of ETTs, the coefficients of the parabolic functions of the nonlinear approximation for the extended Rohrer’s equation for all investigated LMA sizes are listed in Online Supporting Information Table S1. Inertance was of insignificant relevance for all sizes (Inertance ≤ 0.07 cmH_2_O s^2^ l^− 1^). According to the typical flow range predefined by the LMA manufacturer, the pressure drop ranged from 0.4 to 1.5 cmH_2_O (Table 2) and was below the specified maximum pressure drop documented in the datasheets^17^.

**Table 2:** Pressure-drop of LMAs.

LMA size	#1	#1.5	#2	#2.5	#3	#4	#5	#6
Flow [l s^− 1^]	0.25	0.25	0.5	0.5	1.0	1.0	1.0	1.0
ΔP [cmH_2_O]	1.0	0.4	0.6	0.5	1.5	0.9	0.6	0.4
ΔP_manu_ [cmH_2_O]	< 1.2	< 0.8	< 1.0	< 0.8	< 2.0	< 1.2	< 0.8	< 0.5

Pressure-drop (ΔP) of different laryngeal masks at their typical flow predefined by the manufacturer. Results of the study (ΔP) compared to the pressure-drop specified by the manufacturer (ΔP_manu_). LMA = laryngeal mask, # = size.

## Resistance of the laryngeal mask airway

**Fig. 3:**
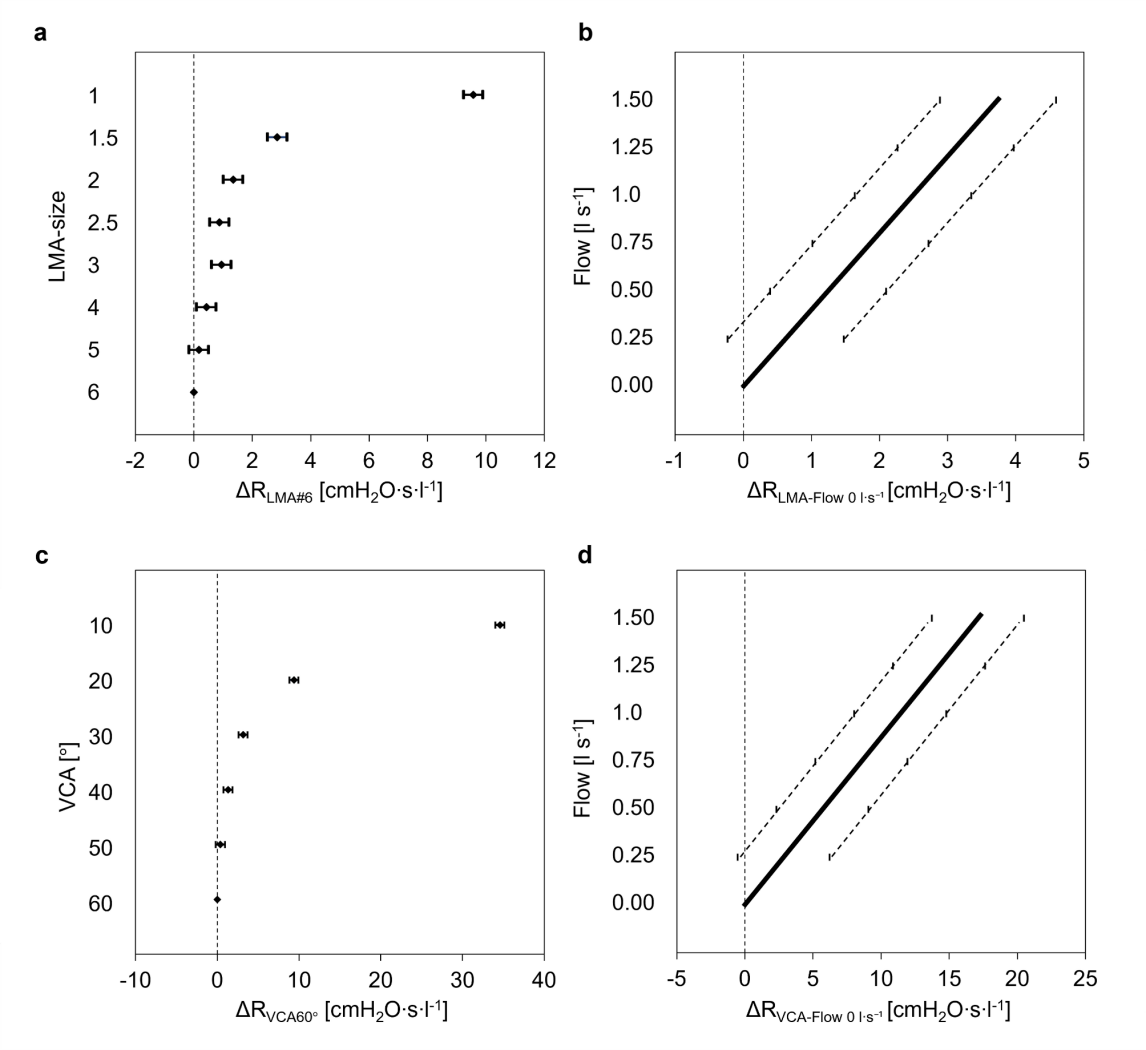
Dependencies of mean resistance. (**a**) Differences of mean resistance (diamonds) of the LMA-sizes compared to LMA#6 (ΔR_LMA#6_) with corresponding 95% confidence intervals (whiskers). (**b**) Differences of mean resistance (bolt line) of the LMAs in dependence of flow compared to flow rate 0 l s^−1^ (ΔR_LMA-Flow 0 l s−1_) with corresponding 95% confidence intervals (doted lines). (**c**) Differences of mean resistance (diamonds) of the different vocal cord angles compared to VCA60° (ΔR_VCA60°_) with corresponding 95% confidence intervals (whiskers). (**d**) Differences of mean resistance (bolt line) of the vocal cords angles in dependence of flow compared to flow rate 0 l s^−1^ (ΔR_VCA-flow 0 l s−1_) with corresponding 95% confidence intervals (doted lines). Mean resistance was calculated by averaging resistances of (**a**) one LMA-size or (**c**) one VCA over the flow range between 0 and 1.5 l s^−1^ in steps of 0.25 l s^−1^ or by averaging resistances of (**b**) all LMA-sizes and (**d**) all VCAs at different flow rates.

The resistance of the LMA was influenced by flow rate (*p* < 0.001) and LMA size (*p* < 0.001). An increase in the flow rate and a decrease in the LMA size increased the resistance (Fig. 3A + B). The mean resistance increased by 1.3 cmH_2_O s l^− 1^ (95%CI 0.3–2.2; *p* ≤ 0.009) per 0.5 l s^− 1^ increase in flow rate. The mean resistances between LMA#4, LMA#5, and LMA#6 (all *p* ≥ 0.157), and between LMA#2, LMA#2.5, and LMA#3 (all *p* ≥ 0.064) were comparable, whereas the mean resistances of LMA#1 and LMA#1.5 differed significantly from all other sizes (all *p* ≤ 0.001).

**Fig. 4:**
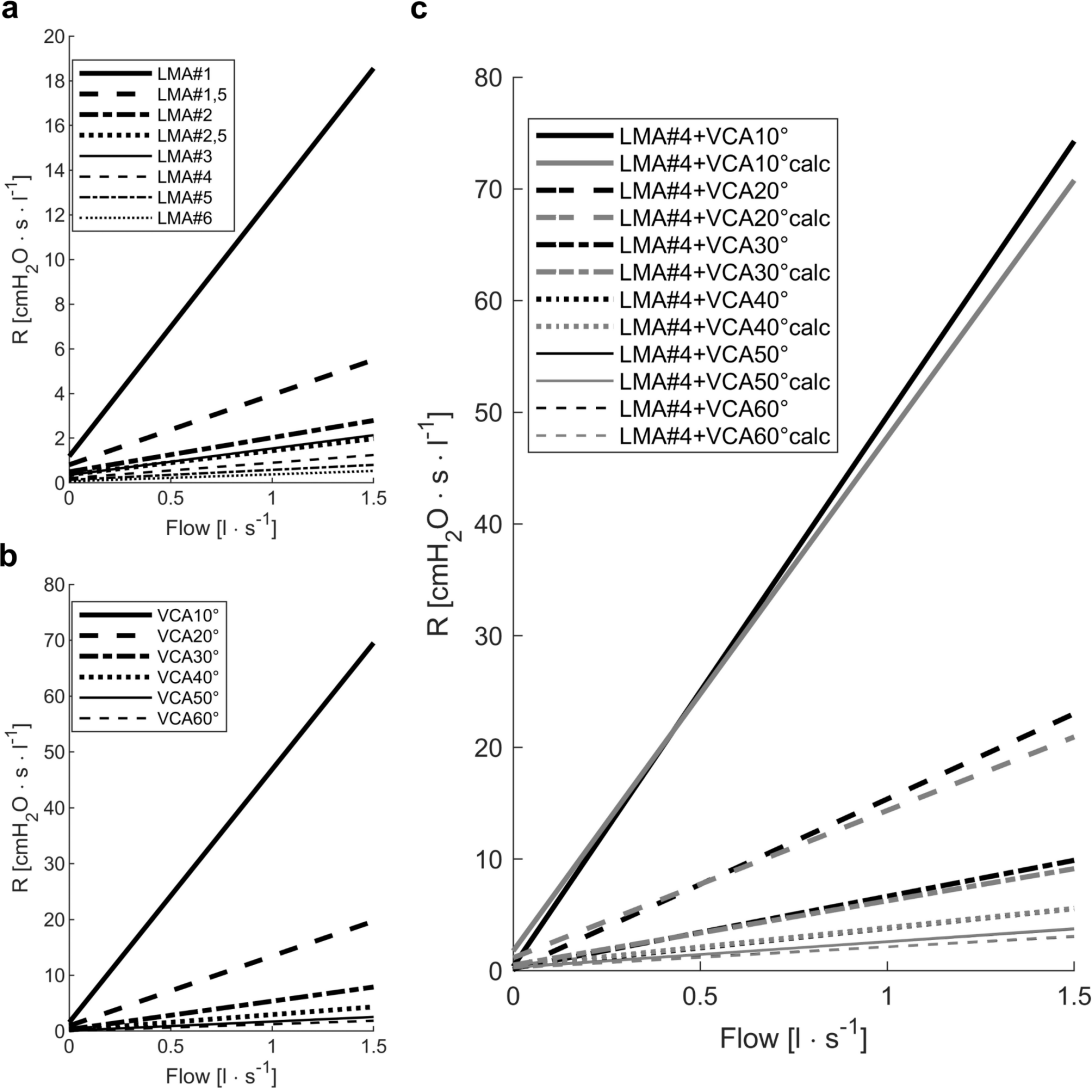
Resistance diagrams. (**a**) Resistance (R) of different sizes of laryngeal mask airways (LMA#) and (**b**) different vocal cord angles (VCA) in dependence of flow. (**c**) Combined resistance of laryngeal mask airway size 4 (LMA#4) and different vocal cord angles (VCA) in black. Grey lines represent the results of the mathematical summation of the single resistance of LMA#4 and the corresponding VCA (calc). Note the completely overlaying graphs of measured and calculated resistances for vocal cord angles higher than 30°.

At a flow rate of 1 l s^− 1^ resistance of LMA#1 (12.8 ± 0.9 cmH_2_O s l^− 1^) was 34-fold higher than that of LMA#6 (0.4 ± 0.4 cmH_2_O s l^− 1^; *p* < 0.001; Fig. 4A). Inspiratory resistance at a flow of 1 l s^− 1^ was higher than expiratory resistance in all LMA sizes (all *p* < 0.001), except for LMA#1 (*p* = 0.986).

### Resistance of the laryngeal model

The resistance of the laryngeal model was influenced by the flow rate (*p* < 0.001) and VCA (*p* < 0.001). Higher flow rates and smaller VCA were associated with higher resistance (Fig. 3C, D). The mean resistance increased by 5.7 cmH_2_O s l^− 1^ (95%CI 2.3–9.1; *p* ≤ 0.021) per 0.5 l s^− 1^ increase in flow rate. The mean resistances of the VCAs between 10° and 50° differed significantly (all *p* ≤ 0.023). The mean resistance of the VCA of 50° was comparable to that of the VCA of 60° (*p* = 0.183).

At a flow rate of 1 l s^− 1^ a VCA 10° (46.8 ± 1.4 cmH_2_O s l^− 1^) ` than a VCA 60° (1.3 ± 0.03 cmH_2_O s l^1^, *p* < 0.001, Fig. 4B). Expiratory resistance at a flowrate of 1 l s^− 1^ exceeded inspiratory resistance at all VCAs (all *p* < 0.001).

## Combined resistance

The combined resistance of LMA#4 and each VCA depended on the flow rate (*p* < 0.001) and VCA (*p* < 0.001). The mean combined resistance increased by 6.6 cmH_2_O s l^− 1^ (95%CI 2.3–10.9; *p* ≤ 0.003) per 0.5 l s^− 1^ increase of flow rate. A VCA ranging between 10° and 30° generated a distinct variation in the combined resistance (*p* < 0.001), whereas a VCAs between 40° and 60° did not result in significant differences in the combined resistances (*p* ≥ 0.188).

**Fig. 5:**
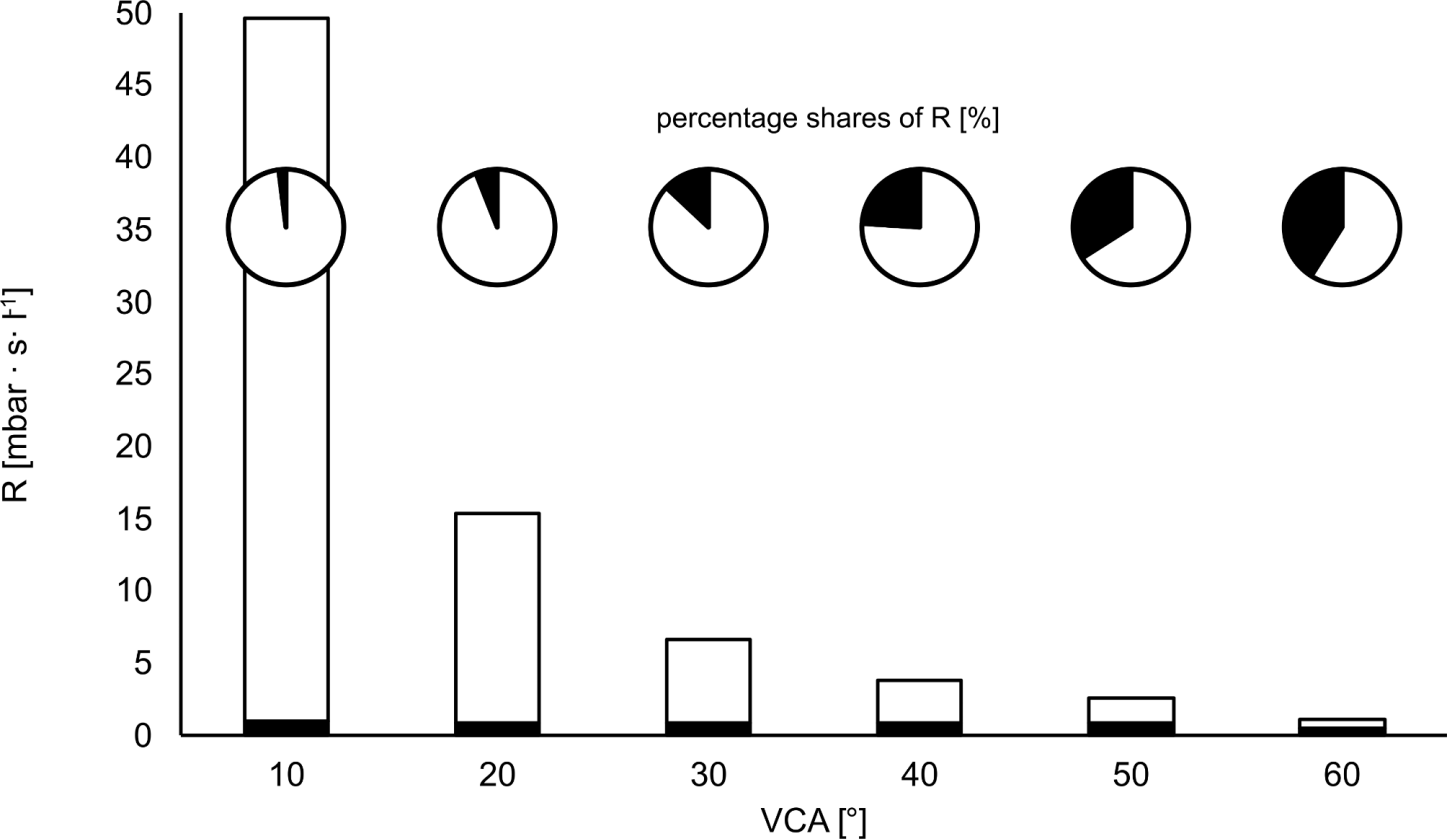
Combined resistance and its percentage shares. Combined resistance (R) of a laryngeal mask airway size 4 (black) and artificial vocal cords (white) with different vocal cord angles (VCA) at a flow rate of 1 l s^−1^. Bars represent the total amount of the combined resistance, pie charts show the percentage shares of the combined resistance.

At a flow rate of 1 l s^− 1^ the main determinant of combined resistance is the VCA, resulting in a 23-fold higher combined resistance with a VCA of 10° (49.6 ± 0.3 cmH_2_O s l^− 1^) compared to a VCA of 60° (2.1 ± 0.1 cmH_2_O s l^− 1^, *p* < 0.001). The LMA contributed between 2 and 41% to the combined resistance whereas the vocal cord angle was responsible for 59–98% of the upper airway resistance (Fig. 5).

The combined resistances were comparable to the sum of the single resistances of LMA#4 and the resistances of the artificial vocal cords with the corresponding VCAs (all *p* ≥ 0.437, Fig. 4C).

## Discussion

The main findings of this investigation can be summarized as follows: (a) The LMA and larynx showed nonlinear, flow-dependent resistance. (b) A smaller LMA size, a smaller VCA, and an increase of flow substantially increase the resistance. (c) As expected, the resistances of the LMA and larynx combine to the upper airway resistance in a summative way. The VCA represented the crucial component of the combined resistance in an adult airway managed with an LMA.

Only a few studies have investigated the resistance of LMAs, and, to our knowledge, a systematic analysis of the resistance of LMAs in contemporary use is lacking. However, systematic analyses are required to understand the resistive behavior of the respiratory system during use of an LMA. Furthermore, to compensate resistance of LMAs during spontaneous breathing or to compare the superimposed WOB of LMAs with other airway devices, systematic knowledge of resistance is crucial. Therefore, we analyzed the resistances of different sizes of LMA and different VCAs at different flow rates as well as their functional interactions using a physical model. In our systematic analysis, we demonstrated that LMA size is a factor in determining resistance, although the anesthesiologist’s scope for size selection is limited by the manufacturer’s size recommendations and the patient’s anatomy. The device resistance increased with a decrease in the internal diameter. This could be explained by the basic relationship between the resistance and diameter of pipes, as described by the Hagen–Poiseuille equation. Analogous coherence was also shown in reinforced LMAs^18^, however comparing the resistance of reinforced LMAs to our results, the same LMA size of a reinforced LMA had a higher resistance because of the distinctly reduced effective inner diameter resulting from internally seated wires. Furthermore, additional modifications of the LMA’s air-conducting system, such as the vertical aperture bars of the LMA classic, could further increase the resistance^19^. Beyond the inner diameter, the flow rate was the main factor determining the resistance of the LMA. The causative mechanism for this nonlinear increase of the pressure-drop over the LMA is the general predominance of turbulent flow in artificial airways, resulting in a nonlinear relationship between resistive pressure-drop and flow rate^20^. Irrespective of the ventilation mode, increasing the flow rate results in a distinctly increase in resistance. The flow-dependent behavior of resistance of LMAs was also described by Reissmann et al. when they compared resistance of ETTs with an internal diameter of 7.5 mm and 8.5 mm to a size 4 LMA^6^. Regarding the different sizes of LMAs in our investigation, the resistance fell clearly below those of the corresponding ETTs. The smallest investigated LMA (LMA#1) came with a resistance, comparable with a ETT of 6.0–6.5 mm of internal diameter^15^ and resistances of LMAs used in adults were lower than those of large tracheostomy cannulas^3^. However, a meaningful comparison of the resistance of the respiratory system of ETTs to LMAs requires consideration of the resistance of the larynx. In awake, spontaneously breathing patients, laryngeal resistance is considered to generate 25–50% of upper airway resistance (0.5–1 cmH_2_O s l^− 1^).^21^ Thereby, laryngeal resistance is highly variable, even in anesthetized and mechanically ventilated patients, who received neuromuscular blocking agents^6^. Moreover, in vivo determination of the resistance in anaesthetized patients is challenging, because placement of a subglottic catheter for pressure measurement is difficult in this highly versatile part of the upper airway, and catheters could provoke a reflex response of the vocal cords, affecting resistance^22,23^. Therefore, we systematically investigated laryngeal resistance in a simplified physical model. The resistance of the laryngeal model depended on both the VCA, resulting in different cross-sectional areas of the glottic opening, and the flow rate, as described by Baier et al.^22^. Despite disregarding the three-dimensional structure along the airstream and the complex geometry of the human glottic opening in our physical model, the resistance of our artificial larynx was comparable to that of in vivo measurements of spontaneously breathing subjects^22^, which emphasizes the importance of the diameter of the cross-sectional area, where the air flows through, and the predominance of turbulent airflow.

During airway management with an LMA, device resistance and laryngeal resistance merge to the intrinsically linked combined resistance, dominating respiratory mechanics. The combined resistance of our model showed flow-dependent, nonlinear, and highly versatile behavior, composed of the sum of the single resistances of the LMA and larynx, following the basic physical principle of serial resistances. Consistent with the findings of in vivo studies^6,19^, VCA formed the dominant part of the combined resistance.

This variability in the combined resistance and its composition of LMA and VCA is of fundamental importance in the understanding of respiratory system mechanics during LMA use. Anesthetists focus on achieving a low combined resistance, first, to facilitate positive pressure ventilation with low peak airway pressures to reduce the risk of oral leakage or gastric insufflation^24^, second, to reduce WOB in spontaneous breathing patients and third, to afford a prompt complete expiration and avoid dynamic hyperinflation^11^. Here, our investigation could provide a better understanding of the mechanics of the respiratory system on an LMA-secured airway and of the measures that were used to reduce resistance of the respiratory system. Due to the negligible low resistance of the LMA itself, providing a wide VCA with an adequate depth of anesthesia or the additive application of neuromuscular blocking agents in laryngospasm seems to be a rational measure to lower the combined resistance. Furthermore, according to the flow dependency of the combined resistance, gentle manual ventilation with low flow rates, especially during narrow vocal cord positions, such as caused by beginning laryngospasm, is a meaningful method to maintain minimal oxygenation and ventilation according to our results. Additionally, with our systematic data of the resistance of different LMA sizes an “automatic LMA compensation” like the automatic tube compensation of some ventilators, could be facilitated to compensate at least the resistance of the LMA in a spontaneous breathing patient, although compensating the combined resistance seems to be challenging due to the variable laryngeal resistance.

Finally, regarding the debate about the ideal airway device that provides the smallest airway resistance, our study, interpreting and transferring the results of a physical model to patients with utmost caution, demonstrated that a universal answer could not be given. The resistance at a flow rate of 1 l s^− 1^ of LMA#4 with a VCA of 30° was comparable to the resistance of a corresponding ETT with an internal diameter of 7.5 mm. However, the combined resistance of an LMA-secured airway could range between superiority and inferiority compared to the resistance of a corresponding ETT, and the entire spectrum could be covered by a single patient, due to the variability of the laryngeal resistance.

As a limitation, the results of our study were based on a physical model, representing a simplified three-dimensional model derived from the human upper airway, consisting of dry and rigid plastic and ignoring the wide interindividual and gender-specific variation of vocal fold length and geometric variances. Interactions between the air and humidified airway mucosa or oscillations of the vocal cords could not be simulated and the complex geometric correlations between vocal fold length, its opening angle in combination with false vocal folds and the geometry of the arytenoid cartilages were simplified considerably to focus on the effect of the cross-section of the glottis and its dependency on the vocal fold angle. It is to emphasize that laryngeal resistance is influenced by a broad range of interindividual and gender specific anatomic variations. Therefore, laryngeal resistance must always be considered individually. However, comparing our results with those of in vivo studies^6^, resistances were in a comparable range, emphasizing the fluid dynamic effects of the flow through the cross-sectional area as the major factor in determining resistance.

An advantage of analyzing combined resistance in a physical model is that subordinate factors influencing resistance, such as the suboptimal position of the LMA, can be minimized. However, this does not reflect the clinical reality, where the position of the LMA, as a device inserted without visual control, is widely variable and malposition could lead to increased combined resistance^6,11^.

The epiglottis was not included in our model. Although projection of the epiglottis on the distal opening of the LMA in the endoscopic view was rarely associated with increased resistance, the additional resistive effect of the epiglottis should not be ingnored^6^. Therefore it must be addressed that combined resistance in daily practice may exceed our results.

Furthermore, the in vivo resistance of the LMA could differ from in vitro measurements because of warming of the LMA to body temperature or because of configurational changes occurring when the LMA adapts to the pharyngeal anatomy after positioning^19^. We investigated a 1st generation LMA, although 2nd generation LMAs are gaining widespread acceptance because of their advantages in terms of leak pressure and their additional drainage channel. However, we made conscious decision to use this type of LMA to investigate a simple geometry and eliminate possible confounders, such as the drainage channel. Thus, we were able to generate meaningful and consistent data about the basic physical principles in our basic scientific investigation. A comparable behavior of resistance of 2nd generation LMAs is likely due to comparable physical boundary conditions.

## Conclusion

The LMA and larynx demonstrate a nonlinear flow-dependent resistive behavior, which is mainly affected by their geometric properties. During anesthesia with an LMA, the patient or ventilator must overcome the combined resistance resulting from the device itself and the larynx. In adults, the main factor of the combined resistance is the highly variable laryngeal resistance, which is determined by the angle of the vocal cords. During the use of an LMA, anesthetists should recognize laryngeal resistance as the main factor determining upper airway resistance and measures during perioperative increase of airway resistance should focus on increasing the cross-section of the glottic opening. In case of an increase of airway resistance, we recommend endoscopic verification of the position of the vocal folds and the placement of the laryngeal mask to address the determining factors swiftly during anesthesia. Further investigations analyzing the resistive properties of the larynx and its susceptibility in vivo are needed to determine the combined resistance in clinical practice.

## Electronic supplementary material

Below is the link to the electronic supplementary material.

Supplementary Material 1

Supplementary Material 2

Supplementary Material 3

Supplementary Material 4

Supplementary Material 5

Supplementary Material 6

Supplementary Material 7

## Data Availability

The data that support the findings of this study are available from the corresponding author, [J.H.], upon reasonable request.
