# Supplementary material for: Upper airway resistance during use of a laryngeal mask airway is flow-dependent and dominated by the laryngeal resistance
Source: Sci Rep. 2024 Oct 9;14:23585. doi: 10.1038/s41598-024-73844-4 (PMC11464783; doi:10.1038/s41598-024-73844-4)
Supplement: Supplementary file 1 — Supplementary Material 1 [file 41598_2024_73844_MOESM1_ESM.docx]

**Supporting information**

**Figure S1:** Picture of the experimental platform. (a) Experimental setting for the measurement of the pressure-drop of the LMAs and VCAs. (b) Experimental setting for the measurement of the combined resistance during simulation of mechanical ventilation with a lung model. (c+d) Method of fixation of the LMA to the contact surface with adhesive tape. P indicates pressure transducers for airway pressure (P_aw_), laryngeal pressure (P_lx_) and tracheal pressure (P_trach_). V̇ indicates pneumotachograph.

**Figure S2:** LMA contact surface. Construction plan of the LMA contact surface. Millimetre is the unit of measurement.

**Figure S3:** Artificial trachea. Construction plan of the artificial trachea. Millimetre is the unit of measurement.

**Figure S4:** Artificial vocal cords. Construction plan of the artificial vocal cords. Millimetre is the unit of measurement.

**Figure S5:** Laryngeal model. Construction plan of the assembled model. Millimetre is the unit of measurement.

**Table S1:** Coefficients for the expanded Rohrer’s equitation. Coefficients of the parabolic functions for all investigated LMA sizes of the nonlinear approximation for the expanded Rohrer’s equitation ($\Delta p=K_{1}\cdot\dot{V}+K_{2}\cdot\dot{V}^{2}+I\cdot\ddot{V}$), separately for inspiration and expiration under ambient conditions. LMA = laryngeal mask, # = size, K_1_i = inspiratory linear coefficient, K_2_i = inspiratory non-linear coefficient, K_1_e = expiratory linear coefficient, K_2_e = expiratory non-linear coefficient, I = inertance.
