# Supplementary material for: Upper airway resistance during use of a laryngeal mask airway is flow-dependent and dominated by the laryngeal resistance
Source: Sci Rep. 2024 Oct 9;14:23585. doi: 10.1038/s41598-024-73844-4 (PMC11464783; doi:10.1038/s41598-024-73844-4)
Supplement: Supplementary file 2 — Supplementary Material 2 [file 41598_2024_73844_MOESM2_ESM.docx]

Table_S1

|  | Inspiration | |  | Expiration | |  |  |
| --- | --- | --- | --- | --- | --- | --- | --- |
|  | K_1_i | K_2_i |  | K_1_e | K_2_e |  | I |
|  | [mbar s l^-1]^ | [mbar s^2^ l^-2^] |  | [mbar s l^-1^] | [mbar s^2^ l^-2^] |  | [mbar s l^-1^] |
| LMA#1 | 1.19 | 11.58 |  | 1.08 | -11.68 |  | 0.067 |
| LMA#1.5 | 0.81 | 3.13 |  | 0.63 | -2.18 |  | 0.043 |
| LMA#2 | 0.49 | 1.53 |  | 0.36 | -1.08 |  | 0.035 |
| LMA#2.5 | 0.37 | 1.07 |  | 0.46 | -0.74 |  | 0.032 |
| LMA#3 | 0.35 | 1.19 |  | 0.45 | -0.79 |  | 0.033 |
| LMA#4 | 0.21 | 0.69 |  | 0.33 | -0.27 |  | 0.027 |
| LMA#5 | 0.14 | 0.45 |  | 0.19 | -0.17 |  | 0.024 |
| LMA#6 | 0.07 | 0.31 |  | 0.15 | -0.13 |  | 0.023 |
