# Supplementary figures and images for: Upper airway resistance during use of a laryngeal mask airway is flow-dependent and dominated by the laryngeal resistance
Source: Sci Rep. 2024 Oct 9;14:23585. doi: 10.1038/s41598-024-73844-4 (PMC11464783; doi:10.1038/s41598-024-73844-4)

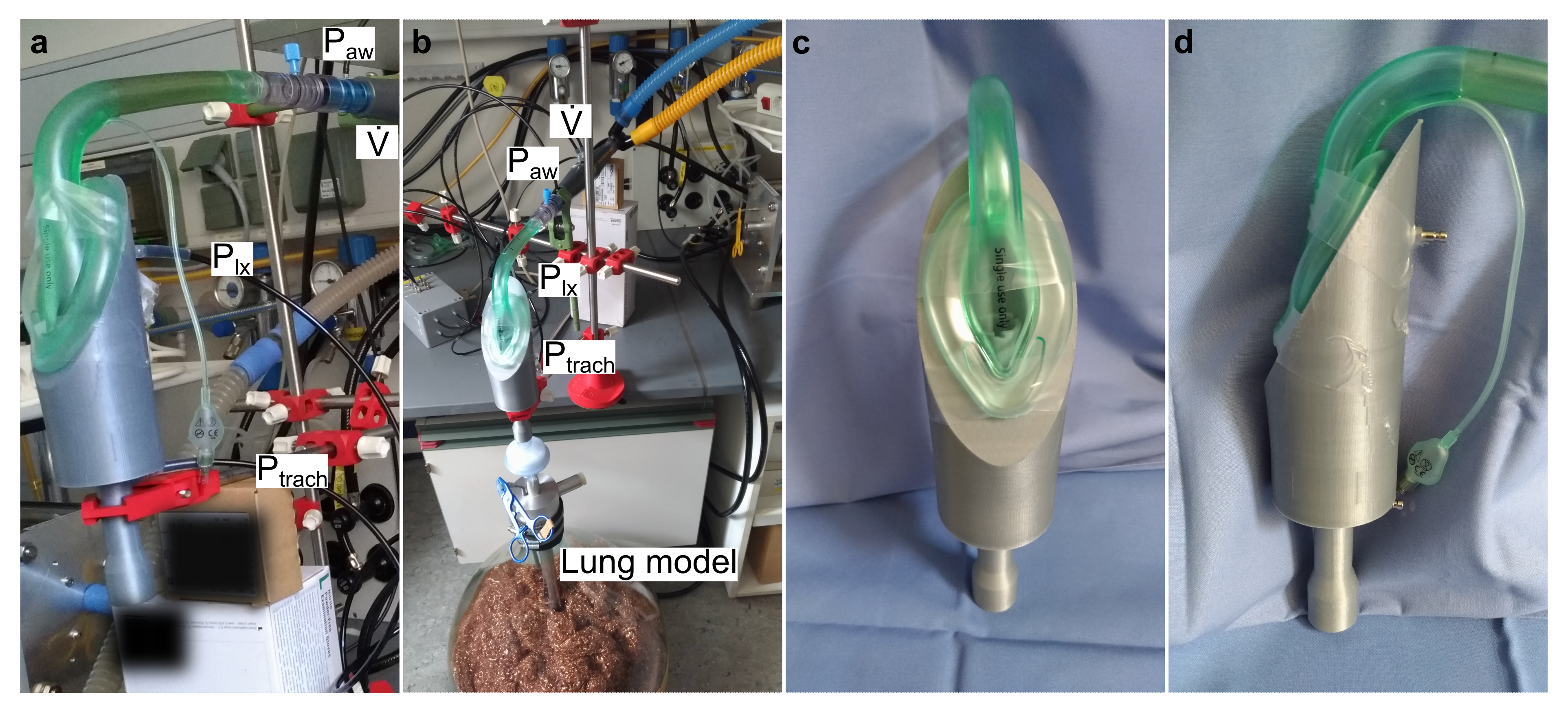

Supplement: Supplementary file 3 — Supplementary Material 3 [file 41598_2024_73844_MOESM3_ESM.tiff]
